# Delaware Dental Association

**Published:** 1867-01

**Authors:** S. Marshall


### DELAWARE DENTAL ASSOCIATION.

At a meeting of the Delaware Dental Association, held in this city, on the 25th inst., the following resolutions were unanimously adopted:—

WHEREAS: We have learned that the "Dental Vulcanite Co.," of Boston, have instituted suits against various Dentists for the use of hard rubber for dental purposes; and believing, as we honestly do, that said Company have no just nor legal claims against Dentists for the use of hard rubber.

*Therefore, Resolved,* That we do not recognize the claims of said Company, and will not accede to their demands until we have done our utmost to see the case prosecuted before the highest legal tribunal of the land; and for that purpose we will unite our energies and our money to enable ourselves and others to legally resist the claims of said Company.

*Resolved,* That we officially inform the several Associations of our action, and publish these resolutions in our city papers and dental periodicals.

*Resolved,* That our executive committee be instructed to correspond with the committee of Philadelphia dentists, and solicit our unity with them and their co-operation with us.

*Resolved,* that our executive committee be requested to solicit subscriptions from the dentists and others of the surrounding country to assist us in our laudable object.

Wilmington, Del., Dec. 26, 1866. S. MARSHALL, Sec'y.

